# Restart of androgen deprivation therapy after goserelin induced pituitary apoplexy in a patient with disseminated prostate cancer a case report and five-years follow-up

**DOI:** 10.1016/j.eucr.2021.101648

**Published:** 2021-03-23

**Authors:** Belal Aljabri, Wolfgang Lilleby, Marta D. Switlyk, Gunnar Tafjord

**Affiliations:** a Department of Oncology, The Norwegian Radiumhospitalet, Oslo University Hospital, Oslo, Norway; b Department of Radiology, The Norwegian Radiumhospitalet, Oslo University Hospital, Oslo, Norway; c Department of Palliative medicine, Akershus University Hospital, Lørenskog, Norway

**Keywords:** Prostate cancer, Pituitary apoplexy, Goserelin, Gonadotropin releasing hormone

## Abstract

Pituitary apoplexy is a clinical syndrome caused by hemorrhage or infarction of a pituitary adenoma. There have been a few reports in the literature of rapid onset of pituitary apoplexy after goserelin injection. To the best of our knowledge, there is no publication in the literature reporting re-introducing goserelin therapy for patients with prostate cancer after the onset of pituitary apoplexy. In this case report, we present the onset and clinico-radiological course of pituitary apoplexy induced by the initiation of goserelin and during continuation of goserelin with up to five-years follow-up.

## Introduction

Androgen deprivation therapy with gonadotropin-releasing hormone agonists (GnRH-a) is a cornerstone in the treatment of patients with metastatic prostate cancer.

Pituitary tumors are common causes of sellar masses but up to 45% of pituitary tumors are reported to be non-functioning, clinically silent adenomas.^1^

Pituitary tumor apoplexy is a rare syndrome that occurs due to hemorrhage or infarction in a preexisting pituitary adenoma.^2^ After the first study in 1994 by Korsic et al.,^3^ there has been a few reports in the literature of rapid onset of pituitary apoplexy after goserelin injection.

In this case we report the clinical experience with re-introducing ADT in a patient who had a goserelin-induced pituitary bleeding and 5 years follow-up.

## Case report

Here, we report a 74-year-old man who underwent primary surgery for prostate cancer (robot-assisted laparoscopic prostatectomy), Gleason score of 6 in 2000. His preoperative prostate specific antigen (PSA) level was 8.8 ng/mL. He was in complete clinical remission until 2010 when a biochemical relapse was confirmed. He underwent salvage radiotherapy to the prostate bed. The patient had a PSA nadir of 1 ng/mL at the end of radiotherapy and was then followed by his local urologist until biochemical progression.

The patient was referred to our hospital after a continuous rise of his PSA level started in 2014. He had no relevant morbidity despite irregular urinary incontinence.

In early 2015, positron emission tomography (PET)/computed tomography (CT) showed a sclerotic lesion in the patient's sternum. The solitary lesion was confirmed by magnetic resonance imaging (MRI) of the spine and thorax. An image-guided biopsy was performed and histology verified metastasis from adenocarcinoma of the prostate.

Because the patient was asymptomatic and the lesion was located close to his heart, radiotherapy was considered to be possibly harmful. In a shared decision the patient was followed by watchful waiting throughout 2015 and regular serum PSA measurements.

Natrium fluoride (NaF) PET/CT follow-up in 2015 showed a new metastatic lesion in the pelvis. The patient remained asymptomatic and watchful waiting was continued until April 2016 when the patient's PSA level increased to 11 ng/mL. Although still asymptomatic the treating oncologist recommended ADT with 50 mg x 1 bicalutamide for 30 days followed by 10.8 mg subcutaneous goserelin (Zoladex®). The first dose was administered in April 2016. Prior to goserelin injection, he had a plasma testosterone level of 7.4 nmol/L.

Two hours after injection, the patient experienced intense headache and vomiting. The symptoms lasted throughout the weekend. The patient was subsequently hospitalized in acute emergency status. While in the emergency care unit, he developed dizziness and double vision.

**Fig. 1:**
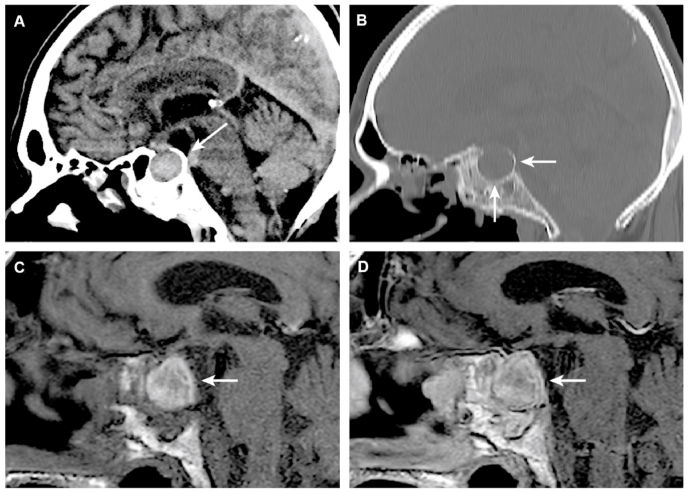
Computed tomography (CT) and magnetic resonance imaging (MRI) findings of pituitary apoplexy after treatment with the gonadotropin-releasing hormone agonists (GnRH-a) for prostate cancer. The hemorrhage has presumably developed in a previously unknown pituitary adenoma. Unenhanced CT of the brain (A, B) shows a large, hyperdense sellar mass (A), consistent with hemorrhage. An enlarged, remodeled sella turcica is noted, secondary to a slow-growing process, presumably macroadenoma (B). Unenhanced (C) and contrast-enhanced T1-weighted MRI (D) at baseline shows a heterogenous, hyperintense mass, consistent with subacute hemorrhage (methemoglobin). The eventual contrast enhancement is difficult to evaluate.

An unenhanced brain CT revealed a large sellar mass and enlarged, remodeled sella turcica (Fig. 1A and B). MRI of the pituitary gland showed a heterogenous tumor that was presumably a macroadenoma with ongoing bleeding (Fig. 1C and D).

Neurosurgical intervention was discussed but declined, and conservative treatment with oral steroids was initiated, resulting in a sudden clinical improvement.

**Fig. 2:**
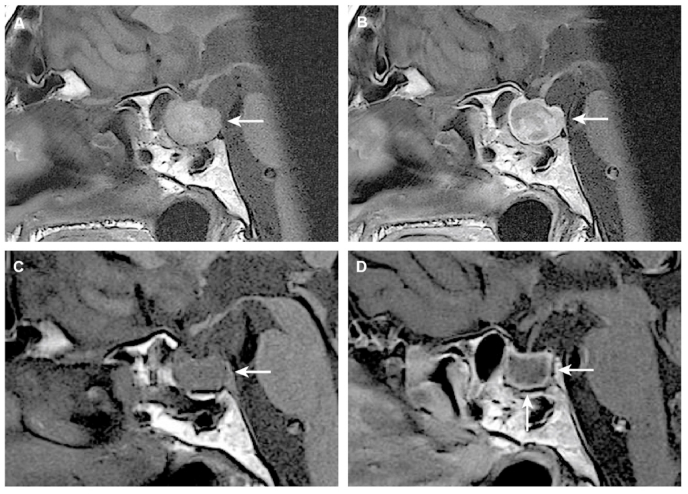
Follow-up magnetic resonance imaging (MRI) scans. MRI performed at the one-month follow-up (A, B) shows partial regression of the sellar lesion. Partial regression of the high signal intensity on T1-weighted MRI (A) and heterogenous, mostly peripheral contrast enhancement (B) is noted, consistent with aging of the blood breakdown products and partial liquefication of the hemorrhage. MRI performed at the one-year follow-up shows chronic, liquefied hematoma with T1-signal characteristics close to water (C) and peripheral contrast enhancement (D).

MRI performed at one-month follow-up showed regression of the lesion (Fig. 2A and B). Further regression was confirmed on MRI at one-year follow-up (Fig. 2C and D). A thorough multi-disciplinary evaluation concluded that rebleeding was unlikely.

The patient was then discharged from hospital with oral cortisone treatment and a follow-up plan involving oncological and endocrinological specialties.

The patient was offered surgical castration but refused. In light of a multi-disciplinary evaluation of a low risk for rebleeding and considering the patient's preference, the responsible oncologist opted for restarting the treatment with 10.8 mg subcutaneous goserelin, a decision the patient agreed to.

The patient received a second goserlin injection in July 2016 at three-month intervals. Biochemical analysis in August 2016 showed a decline in the PSA level to 0.64 ng/mL and in testosterone to <0.4 nmol/L.

Laboratory analysis showed decreased estradiol, follicle stimulating hormone (FSH) and luteinizing hormone (LH) values indicating a partial frontal lobe pituitary gland failure. Prolactin, growth hormone, adrenocorticotropic hormone (ACTH) and thyroid-stimulating hormone (TSH) were within normal levels.

No side effects were observed while the patient's PSA level continued to decline and remained low (<0.03 at last control in March 2021), with a therapeutic testosterone castration level (<0.4 nmol/l) under hormone treatment with goserelin injection.

In parallel, the patient continued cortisone and later thyroxin replacement therapy under close observation. Last endocrinological control in November 2020 showed well regulated values. Last MRI of pituitary gland (02.09.20) showed no pathology.

## Discussion

We reported here the case of a patient with sudden headache related to pituitary apoplexy after injection of goserelin for treatment of his metastatic PC.

The mechanism of pituitary apoplexy remains unknown but it is believed that a sudden increase in sellar content compresses the surrounding structures and portal vessels resulting in acute, severe headache, visual disturbances, and impairment of pituitary function.^4^

To best of our knowledge there is a lack of clinical experience with re-introducing ADT after pituitary bleeding caused by GnRH-agonist. Our patient recovered from pituitary apoplexy and had no further complications related to reintroduction of goserelin by the time of submitting this report almost five years after reintroducing ADT. Since ADT is known to have other side effects such as sexual impairment and metabolic syndrome, the decision to reintroduce ADT in our patient with a partial pituitary gland failure has been regularly assessed and to date, no additional side effects have occurred.

## Conclusion

ADT aiming to minimize testosterone levels either achieved by orchiectomy or chemically using LHRH-axis manipulation has become the mainstay for treatment of relapsed PC. In view of the relative high incidence of asymptomatic pituitary mass, cerebral complications may be underreported in men treated with ADT. This case report highlights that long-term stable disease could be achieved in a metastatic patient by thoroughly re-starting ADT despite of his history of pituitary apoplexy.
